# Short Communication: Health Interventions in Volcanic Eruptions—Community Wearability Assessment of Respiratory Protection against Volcanic Ash from Mt Sinabung, Indonesia

**DOI:** 10.3390/ijerph15112359

**Published:** 2018-10-25

**Authors:** Karen S. Galea, Judith Covey, Sari Mutia Timur, Claire J. Horwell, Fentiny Nugroho, William Mueller

**Affiliations:** 1 Centre for Human Exposure Science, IOM, Research Avenue North, Riccarton, Edinburgh EH14 4AP, UK; will.mueller@iom-world.org; 2 Science Labs., Department of Psychology, Durham University, South Road, Durham DH1 3LE, UK; j.a.covey@durham.ac.uk; 3 Yakkum Emergency Unit (YEU), Jl. Kaliurang KM 12, Dusun Candi 3, no. 34, Desa Sardonoharjo, Ngaglik, Sleman, Yogyakarta 55581, Indonesia; sari.mutiatimur@gmail.com; 4 Department of Earth Sciences, Institute of Hazard, Risk & Resilience, Durham University, Lower Mountjoy, South Road, Durham DH1 3LE, UK; claire.horwell@durham.ac.uk; 5 Department of Social Welfare Faculty of Social and Political Science, University of Indonesia, Kampus Baru UI Depok, Jawa Barat 16424, Indonesia; fentiny2015@gmail.com

**Keywords:** facemask, ash, volcano, wearability

## Abstract

Inhalation of ash can be of great concern for affected communities, during and after volcanic eruptions. Governmental and humanitarian agencies recommend and distribute a variety of respiratory protection (RP), commonly surgical masks but, also, industry-certified N95-style masks. However, there is currently no evidence on how wearable they are within affected populations or how protective wearers perceive them being against volcanic ash (which will influence the likelihood of uptake of recommended interventions). Volunteers living near Mt. Sinabung, Sumatra, Indonesia, participated in a field wearability study, which included a high-efficiency mask certified to industry standards (N95-equiv.); a standard, pleated surgical mask (Surgical); a Basic flat-fold mask (Flat-fold), and the surgical mask plus a scarf tied over the top (Surgical Plus) to improve fit. These types of RP had all performed well during earlier laboratory filtration efficiency tests. The N95-equiv. mask had performed significantly better than the other RP in the subsequent total inward leakage volunteer trials, whilst the Flat-fold and Surgical masks performed poorly, letting in a third of PM_2.5_ particles (data published elsewhere). Thirty volunteers wore each mask for a 15-min walk before being asked to rate the comfort, breathability and perceived protection and fit of each. After wearing all of the masks, volunteers compared and identified their preferred type of protection. The feedback received from the volunteers suggested that the Surgical Plus and N95-equiv. masks were rated as being significantly hotter and more humid than other masks. The Flat-fold was rated to have better breathability than the other masks. The N95-equiv. mask was ranked as providing the best level of effectiveness of the four masks tested. Ultimately, when asked which type of mask they would choose to wear during ashfall, 33% selected the Flat-fold mask due to its comfort and simplicity, with the Surgical Plus being the least likely to be chosen of the four tested. The study findings are of benefit to agencies who need to make informed decisions on the procurement and distribution of RP for use by those affected in future eruptions and the provision of advice to communities on their usage.

## 1. Introduction

Exposure to volcanic ash has been shown to exacerbate respiratory conditions and increase hospital visits [1]. A range of respiratory protection (RP) is recommended and distributed by agencies to help protect affected citizens from inhaling ash during volcanic crises [2]. The effectiveness of RP is dependent on several factors: (i) the design, to prevent penetration of a specific hazardous substance; (ii) reduction of exposure to the level required to protect the wearer’s health; and iii) appropriateness for the individual wearer, task, and environment [3].

In our previous research, Mueller et al. [4] reported the filtration efficiency (FE) of 17 types of RP typically used during volcanic crises, tested in a laboratory setting. The median FE of individual RP types for two volcanic ashes, combined, ranged considerably from 23.4% for a veil from Indonesia to 99.8% for an industry-certified FFP2 (N95-equiv.) mask [5]. Surgical masks, which were distributed following the eruption of Kelud volcano in Indonesia in 2014 [6] had median FEs of 98.4% and a ‘flat-fold’ mask also distributed in Indonesia by the Red Cross had a median FE of 98.1%. Steinle et al. [7] reported the total inward leakage (TIL) of the four best performing masks (plus, a surgical mask was tested with an added layer of material tied over the top) from Mueller et al. [4], which was assessed in a laboratory volunteer simulation study. The N95-equiv. mask was reported to perform significantly better, (mean TIL 9%), compared to the other masks tested (>22% TIL), including the Surgical and Flat-fold masks, which both had TIL of 35%. The volunteers also reported on the wearability and perceived effectiveness of the masks tested. The N95-equiv. mask was perceived to provide the best protection of the tested masks, but also was perceived as being uncomfortable and more difficult to breathe through. The Flat-fold and Surgical masks were both perceived to be the worst in terms of protection, as they were thought to have lots of gaps and a poor fit, but to be the most comfortable and breathable.

Whilst the results of the laboratory-based studies provided valuable information on the efficacy of RP typically used against volcanic ash, and their perceived effectiveness and wearability, there is still a need to consider their acceptance and perceived performance in real-life settings by those residing in communities affected by volcanic ash. Mount Sinabung, a volcano in North Sumatra, Indonesia, has been continuously active since September 2013, producing extensive volcanic ash clouds. These conditions provided a timely opportunity to test the wearability of RP used during volcanic eruptions, in situ (in a hot, humid environment), for the first time.

The aim of the current study was to identify which of the masks previously assessed in the laboratory setting [7], were perceived to be most wearable by residents living in communities affected by the Sinabung eruptions, based on criteria such as comfort, fit provided, and the ease of breathing when worn, in addition to perceived effectiveness.

## 2. Methods

### 2.1. Volunteer Recruitment

A convenience sample was obtained of 30 volunteers aged between 18 and 65, with a mix of ages and gender, who resided in areas affected by the Mount Sinabung volcanic ash. Potential volunteers were excluded from participation, based on self-reports, if they were outside the 18–65 age range, had cardiovascular or respiratory problems (e.g., asthma), suffered from claustrophobia, had mobility issues or, in the case of female volunteers, were pregnant or breastfeeding. The volunteers, based on self-reports, were to be fit enough to walk for about an hour (4 × 15-min with short breaks) at a comfortable pace. Volunteers provided informed, written consent, and were informed that they could leave the study at any time without giving a reason. At the end of the study, the volunteers had the option of retaining the masks they had tested (along with a small cloth bag to keep them in) to compensate them for their time and any inconvenience caused.

### 2.2. Respiratory Protection Selection

**Figure 1:**
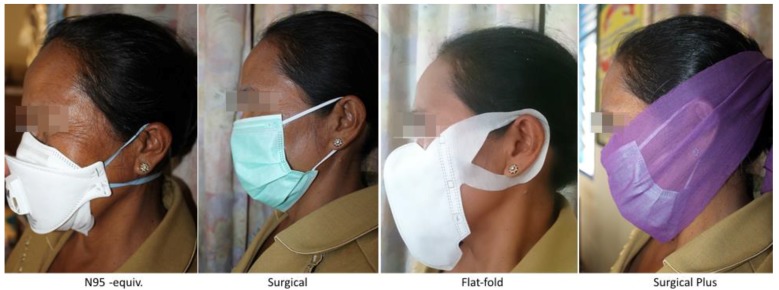
Selected masks for testing in the wearability study.

Mueller et al. [4] and Steinle et al. [7] reported the best performing masks in laboratory-based studies, with key results being summarised in the Introduction of this manuscript. From these tests, three masks were selected for testing in the current wearability study (Figure 1):

1. An industry-certified (EN 149: 2001 standard) 3M Aura 9322 FFP2 respirator (N95-equiv.) (hereafter referred to as N95-equiv.) fitted with a valve to release exhaled, humid air.
2. Standard, pleated surgical mask with ear loops (hereafter referred to as the Surgical).
3. Basic flat-fold mask as supplied by PMI (Indonesian Red Cross, but manufactured in Japan) (hereafter referred to as the Flat-fold).

In addition, the surgical mask was tested in combination with an intervention (a scarf, also shown in Figure 1) (hereafter referred to as the Surgical Plus), as Steinle et al. [7] had shown that adding an extra layer (they used a bandage) improved the fit and, therefore, the effectiveness of these masks. Steinle et al. [7] also tested a Japanese mask marketed for PM_2.5_ exposure reduction. This mask was not tested in this study as they are not available outside of Japan.

### 2.3. Experimental Setup

Villages were chosen for recruitment depending on where ash was falling, based on local judgment and due consideration of the wind direction. These included the villages of Payung, Ndokum Siroga, Sigarang-garang, Tiga Kicat, and Tiga Pancur. Upon recruitment, volunteers were asked to complete an interviewer-led questionnaire, which included basic demographic questions (e.g., age, gender, educational level, employment). In addition, they were asked whether they had previously worn any type of mask/facial protection to protect themselves from ash inhalation and, if so, to provide details regarding the type of mask worn and where it was obtained.

The order of wearing the masks was randomly determined for each volunteer. The researcher then provided the volunteer with the first allocated type of mask to wear. The volunteer fitted the mask themselves without any specific instructions, assistance, or guidance from the researcher.

The volunteers (no more than six at a time) were taken on a 15-min walk on a predetermined route at a comfortable, moderate pace. Researchers periodically checked that the volunteers were comfortable and able to continue during each walk. At the end of the walk, volunteers were asked to rate the comfort, hotness, humidity, breathability, perceived protection level, and fit of the mask worn using a five-point Likert scale (with 1 and 5 representing the least and greatest agreement, respectively, for a given criterion). Volunteers could also provide further contextual information if they chose to do so. After a break, this procedure was repeated for the other three types of masks with the volunteers completing the same set of questions at the end of each walk. Once all four walks had been completed, the volunteers were asked to compare the masks and select their preferred type of protection. The volunteers were also asked to rank, using the above-mentioned criteria, how their usual form of protection, if any, compared to the tested masks.

### 2.4. Ethical Approval

Ethical approval was given by the Ethics Board of the Department of Earth Sciences, Durham University (Ref: ESE20171002CH). Permission for the research was also given by the local Ministry of Home Affairs (Kesbangpol Karo District), Indonesia.

### 2.5. Data Analysis

The questionnaire data were entered into the Jisc Online Surveys (formerly BOS) (2018, Jisc, Bristol, UK) and then imported into IBM SPSS 22 Statistics Software (Version 22, IBM, Armonk, NY, USA) for data analysis. Descriptive statistics of the study population demographic were obtained. Mauchly’s Test of Sphericity and Greenhouse-Geiser corrections were used to assess rankings of each wearability criterion using repeated measures [8], and pairwise comparisons of individual masks were undertaken to evaluate differences between each of the masks. The Bonferroni correction was used for multiple pairwise comparisons. Chi-squared tests were used to assess differences in the selection of the perceived best mask for each criterion.

## 3. Results

### 3.1. Participant Characteristics

Surveys were conducted in Payung village in a schoolyard (15 volunteers) and Ndokum Siroga village (seven volunteers) on 11 December 2017; in Sigarang-garang (three volunteers) and Tiga Kicat (four volunteers) villages on 14 December 2017; and in Tiga Pancur village on 15 December 2017 (one volunteer), totaling 30 volunteers.

The characteristics of the volunteers who participated in the study are detailed in Table 1. All 30 volunteers completed each of the trials for the four masks being tested, resulting in 120 wearability trials being completed. We report the results for the whole study population, rather than by the various characteristics (e.g., gender differences etc.) due to the small numbers within each of these.

### 3.2. Previous Use of Facemasks

All 30 volunteers reported that they had previously used surgical masks when there was ashfall. Half of the volunteers reported obtaining these masks from their community health center, four (13%) reported that they had obtained them from the Indonesian Red Cross, three (10%) from their local government, with the eight (27%) others quoting sources such as their school, civil society organizations and a disaster management agency. One (3%) volunteer advised that they had used an N95-equiv. mask when there was ashfall, obtaining this from the Indonesian National Army. No one advised that they had worn a Flat-fold mask. Eleven (37%) said they wore a surgical mask with another layer (e.g., bandana, scarf or veil over the top).

### 3.3. Mask Ratings

#### 3.3.1. Comfort

According to the ratings for comfort, the Flat-fold mask was given the highest mean score (see Table 2), though there were no significant differences resulting from pairwise comparisons between the individual masks (*p* ≥ 0.084) (Greenhouse-Geisser F = 2.779, *p* = 0.059; see Table 2). The Flat-fold mask was also most frequently ranked first for comfort; as shown in Table 3, 50% of participants ranked the flat-fold mask first for comfort, whereas only 7% ranked the surgical masks first for comfort. 

#### 3.3.2. Hotness and Humidity

The Surgical Plus and N95-equiv. masks were each rated as significantly hotter (*p* ≤ 0.021) than the Flat-fold and Surgical masks (Greenhouse-Geisser, F = 7.818, *p* < 0.001). The N95-equiv. mask was deemed to be more humid than the surgical (*p* = 0.033) and Flat-fold (*p* < 0.001) masks, and the Surgical Plus was rated as more humid than the Flat-fold (*p* < 0.001) mask (Greenhouse-Geisser, F = 3.130, *p* < 0.001). There was no difference in either hotness (*p* = 1.00) or humidity (*p* = 1.00) ratings between the Surgical Plus and N95-equiv. masks or the Flat-fold and Surgical Masks (*p* ≥ 0.104; see Table 2).

#### 3.3.3. Breathability

The Flat-fold mask was rated as easier to breathe with compared to each of the three other types of masks (*p* ≤ 0.042; Greenhouse-Geisser, F = 9.734, *p* < 0.001). The Surgical was rated as significantly easier to breathe with than the Surgical Plus mask (*p* = 0.034), and no significant difference was identified between the Surgical and N95-equiv. masks (*p* = 0.715; see Table 2). The Flat-fold mask was ranked by over half (57%) of the volunteers to have the best breathability (see Table 3).

#### 3.3.4. Mask Adjustment/Fit

At least half the participants needed to adjust the N95-equiv. and Surgical Plus masks when putting on and when wearing. The Flat-fold mask was rated as significantly more difficult to adjust than the Surgical Plus (*p* = 0.011) and N95-equiv. (*p* = 0.027) masks (Greenhouse-Geisser, F = 5.153, *p* = 0.003). There was no significant difference in mean ratings of fit between the four masks (Greenhouse-Geisser, F = 2.647, *p* = 0.070; see Table 2), though the N95-equiv. was selected by over half (57%) as having the best fit (see Table 3).

Eighty and 77% of volunteers reported feeling gaps when wearing the Surgical and Flat-fold masks, respectively, with 40% of the volunteers reporting gaps when wearing the Surgical Plus mask. Only 17% of participants reported to feel gaps when wearing the N95-equiv. mask. When gaps were reported, these were typically under the chin, at the nose area or between the nose and cheek.

#### 3.3.5. Perceived Effectiveness and Overall Preference

The N95-equiv. mask was rated more effective than the Surgical (*p* = 0.007) or Flat-fold masks (*p* = 0.030), but not significantly more than the Surgical Plus (*p* = 0.392). The Surgical and Flat-fold masks were rated as equally effective by the volunteers (*p* = 1.00; Greenhouse-Geisser, F = 4.792, *p* = 0.004; see Table 2). The N95-equiv. was selected by over half (57%) as being the most effective (see Table 3). In terms of overall preference, the Flat-fold mask was selected most frequently (40%; see Table 3).

When asked to select one of the tested masks or their own, the Flat-fold was chosen by a third to be the mask which they would wear during ashfall, with justification including that it was perceived as being comfortable, simple to use, light, not hot/sticky when worn, and that it was considered to provide enough protection. Two volunteers indicated that they would choose to wear their own mask (but did not mention type), with one other volunteer stating that they would use a cloth material.

## 4. Discussion

We present the wearability of four types of RP as tested by volunteers residing in a community affected by volcanic ashfall. These devices were all reported to perform well during earlier laboratory filtration efficiency tests [4]. The N95-equiv. mask also performed well in total inward leakage tests, but the Surgical and Flat-fold masks were substantially less effective on volunteers, although this was improved, somewhat, by adding an additional cloth intervention on top of the Surgical mask [7].

Of the devices tested, the N95-equiv. was perceived by the volunteers to provide the best fit and to be the most effective in providing protection against volcanic ash, both of which were confirmed in the laboratory tests. This is not surprising given that this mask is designed to meet industry standards and has several design features (e.g., two head straps, foamed rim and an adjustable nose clip) to allow the wearer to improve the fit. However, it was also evident that volunteers had concerns about the comfort and ease of breathing when wearing the masks, which echoed the opinions of the laboratory study participants reported by Steinle et al. [7]. Such perceptions could adversely impact user uptake and acceptance of effective RP, as well as its use over longer periods of time. It was evident that only one volunteer reported to have previously used N95-equiv. devices and so there is a lack of familiarity with how these masks ‘feel’ when being worn in comparison to the RP more typically used by the communities. This lack of familiarity could affect user acceptance, which may be minimized through appropriate education and instruction during mask distribution.

This being said, none of the volunteers reported to have previously used a Flat-fold mask (these have previously been distributed in Yogyakarta, Java during eruptions of Merapi volcano, but this is in a different part of Indonesia) and, yet, this was ranked as being the preferred mask of the four tested. Whilst it did not rank as highly in terms of fit and perceived level of protection as the N95-equiv. mask, it was ranked highest with respect to comfort and ease of breathing. These aspects may have influenced the overall ranking by volunteers. This highlights the juxtaposition between people’s perceptions of the importance of comfort over the need for effective protection. The Flat-fold mask, whilst being made of material that is effective at filtering fine particles [4], has no features to secure it to the face, therefore impacting its effectiveness [7]. The respondents’ strong preference for this mask, overall, perhaps is an indication of their level of concern and perceived understanding of the hazard of inhaling ash. If this community had considered the ash to be highly toxic, they may well have ranked a more-effective mask over a more comfortable one.

Surgical masks are thought to be the most distributed intervention in volcanic eruptions in Indonesia [6] (and possibly elsewhere), being distributed by the million by government agencies in Yogyakarta after the 2010 Merapi eruption and the 2014 Kelud eruption. Despite their widespread use, and all of our volunteers reporting to have previously used such RP (and therefore being familiar with them), they were reported to be their least preferred mask of the four tested. This could be because they had been made aware of the need to improve fit by adding an additional cloth layer, and because the quality of the N95-equiv. mask was so immediately apparent. However, this does not explain why the respondents preferred the Flat-fold (which is clearly of lesser quality than the N95-equiv., and has noticeably poorer fit) above the Surgical mask.

The wearability study does has several limitations. Our study was restricted to four types of RP, selected based on their performance in our earlier laboratory studies [4,7]. We therefore cannot comment on the wearability or level of protection of other RP that communities may choose to wear. We did not assess the facial size and shape of the volunteers or establish the actual fit of the RP on their face; however, a range of adult male and female facial shapes and sizes were evident in our study population. We tested the wearability during a short duration walking activity, which cannot represent all activities that an individual may undertake whilst wearing a mask. It is unknown how volunteers’ opinions and perceptions may change over longer periods of use and when undertaking tasks that may result in higher breathing rates. Finally, the actual efficacy of the RP in protecting the volunteers from inhaling volcanic ash was not assessed, as this was not within the remit of the wearability study. Whilst not an actual limitation of our study, we should highlight that we did not aim to assess the monetary or logistical costs associated with the bulk procurement, storage, and distribution of each RP tested, which agencies may also consider.

These limitations, however, do not negate the value of our findings. Our study provides much-needed insight into potential wearability barriers to uptake of the most effective RP interventions. There is evidence that some agencies are moving towards providing RP interventions in eruption crises, based upon ethical principles of providing the most effective protection as opposed to the most easily procured/stockpiled (according to the precautionary principle where something is better than nothing) [9]. Agencies need to be aware that the uptake of RP will be affected by perceptions of comfort and breathability, so accompanying information should be designed to encourage uptake despite comfort issues, especially in locations with hot/humid climates. If this can be achieved, it is more likely that effective masks will be accepted and used by communities affected by eruption (and other particulate pollution) crises.

## Figures and Tables

**Table 1:** Description of the volunteers.

Descriptor	*N*	%
Sex		
Male	16	53
Female	14	47
Occupation		
Teacher	12	40
Farmer Entrepreneur	8 5	27 17
Other ^1^	5	17
Highest level of schooling/education completed		
Less than high school	5	17
At least high school	25	83
	Mean	Range
Age (years) at time of recruitment	40	(19–57)

^1^ Includes midwife, mechanic, volcanology officer, student, cleaner.

**Table 2:** Mean (Standard Deviation) scores (out of 5) for each mask and wearability criteria.

Mask	Wearability Criteria						
	Comfort	Hotness	Humidity	Difficulty Breathing	Ease of Adjustment	Fit	Effectiveness
N95-equiv.	3.0 (1.5)	2.3 (0.8)	2.0 (0.7)	2.8 (1.1)	3.2 (1.0)	3.5 (1.2)	3.3 (1.1)
Surgical	2.9 (1.0)	1.8 (0.6)	1.6 (0.6)	2.4 (1.0)	3.7 (1.0)	3.2 (1.0)	2.4 (0.9)
Flat-fold	3.6 (1.1)	1.5 (0.5)	1.3 (0.5)	1.8 (0.9)	3.9 (0.7)	3.5 (1.2)	2.6 (1.0)
Surgical Plus	2.8 (1.3)	2.3 (1.1)	1.9 (0.7)	3.0 (1.0)	3.1 (1.2)	2.8 (1.2)	2.8 (0.9)

**Table 3:** The percentage (%) of participants ranking each mask first for each wearability criterion ^+^.

Mask	Wearability Criteria				
	Comfort ***	Ease of Breathing ***	Fit ***	Effectiveness ***	Overall **
N95-equiv.	30.0	23.3	56.7	56.7	26.7
Surgical	6.7	20.0	16.7	10.0	13.3
Flat-fold	50.0	56.7	16.7	23.3	40.0
Surgical Plus	13.3	0.0	10.0	10.0	20.0

^+^ The complete ranking for each of the wearability criteria is provided as Supplementary Material; ** *p* < 0.01; *** *p* < 0.001. Note: overall % for Fit totals 100.1 due to rounding.

## Supplementary Materials

The following are available online at http://www.mdpi.com/1660-4601/15/11/2359/s1, Table S1: Percentage (%) of participants ranking each mask first, second, third and fourth for comfort, Table S2: Percentage (%) of participants ranking each mask first, second, third and fourth for ease of breathing, Table S3: Percentage (%) of participants ranking each mask first, second, third and fourth for fit, Table S4: Percentage (%) of participants ranking each mask first, second, third and fourth for effectiveness, Table S5: Percentage (%) of participants ranking each mask first, second, third and fourth for best liked.
