# Supplementary material for: Short Communication: Health Interventions in Volcanic Eruptions—Community Wearability Assessment of Respiratory Protection against Volcanic Ash from Mt Sinabung, Indonesia
Source: Int J Environ Res Public Health. 2018 Oct 25;15(11):2359. doi: 10.3390/ijerph15112359 (PMC6266167; doi:10.3390/ijerph15112359)
Supplement: Supplementary file 1 [file ijerph-15-02359-s001.pdf]

Table S1. Percentage (%) of participants ranking each mask first, second, third and fourth for comfort.

Mask	Participants Mask Ranking (%)			
	First	Second	Third	Fourth
N95-equiv.	30.0	30.0	10.0	30.0
Surgical	3.7	30.3	43.3	20.0
Flat-fold	50.0	26.7	16.7	6.7
Surgical Plus	13.3	13.3	30.3	43.3

Table S2. Percentage (%) of participants ranking each mask first, second, third and fourth for ease of breathing.

Mask	Participants Mask Ranking (%)			
	First	Second	Third	Fourth
N95-equiv.	23.3	6.7	16.7	53.3
Surgical	20.0	53.3	20.0	6.7
Flat-fold	56.7	23.3	16.7	3.3
Surgical Plus	0.0	16.7	46.7	36.7

Table S3. Percentage (%) of participants ranking each mask first, second, third and fourth for fit.

Mask	Participants Mask Ranking (%)			
	First	Second	Third	Fourth
N95-equiv.	56.7	13.3	13.3	16.7
Surgical	16.7	10.0	53.3	20.0
Flat-fold	16.7	53.3	10.0	20.0
Surgical Plus	10.0	23.3	23.3	43.3

Table S4. Percentage (%) of participants ranking each mask first, second, third and fourth for effectiveness.

Mask	Participants Mask Ranking (%)			
	First	Second	Third	Fourth
N95-equiv.	56.7	16.7	3.3	23.3
Surgical	10.0	16.7	46.7	26.7
Flat-fold	23.3	40.0	23.3	13.3
Surgical Plus	10.0	26.7	26.7	36.7

Table S5. Percentage (%) of participants ranking each mask first, second, third and fourth for best liked.

Mask	Participants Mask Ranking (%)			
	First	Second	Third	Fourth
N95-equiv.	26.7	30.0	13.3	30.0
Surgical	13.3	30.0	43.3	13.3
Flat-fold	40.0	26.7	16.7	16.7
Surgical Plus	20.0	13.3	26.7	40.0
